# Supplementary material for: In situ quantification of osmotic pressure within living embryonic tissues
Source: Nat Commun. 2023 Nov 2;14:7023. doi: 10.1038/s41467-023-42024-9 (PMC10622550; doi:10.1038/s41467-023-42024-9)
Supplement: Supplementary file 1 — Supplementary Information [file 41467_2023_42024_MOESM1_ESM.pdf]

Supplementary Information

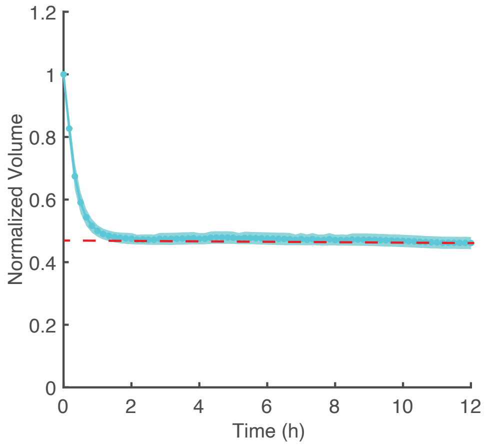

Supplementary Figure 1: Monitoring droplet volume at long timescales. Inner droplet volume normalized to its initial volume as a function of time (initial radius, 38 μm). Double emulsion droplets were placed in cell culture media and monitored for 12h (N=13). After its initial relaxation to its equilibrium value, the equilibrium droplet volume does not show any significant change over these long time periods. Mean ± SD (shown as error band). Source data are provided as a Source Data file.

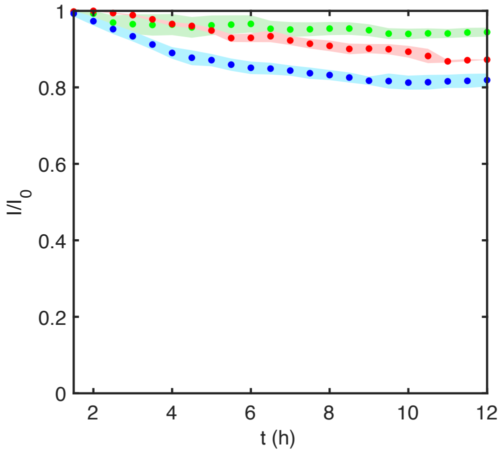

Supplementary Figure 2: Temporal decay in fluorescence intensity in the inner droplet for different laser powers. Temporal decay of the emission intensity I normalized to the initial intentity I_0 for droplets in a 0.4M NaCl solution, for different laser powers: 0.002mW (green; N=6), 0.004 mW (red; N=4), and 0.006 mW (blue; N=5). All other imaging parameters were the same in all cases. After 12h, the inner core intensity is 97% of the maximum intensity at 0.002mW laser power intensity. The reduction in fluorescence intensity after 12h increases with the laser power of excitation illumination, suggesting that the reduction in intensity is caused by photobleaching rather than PEG exiting the inner droplet. Even if there was leakage of PEG, these results indicate that PEG loss would be limited to 3% over 12h. Mean ± SD (shown as error band). Source data are provided as a Source Data file.

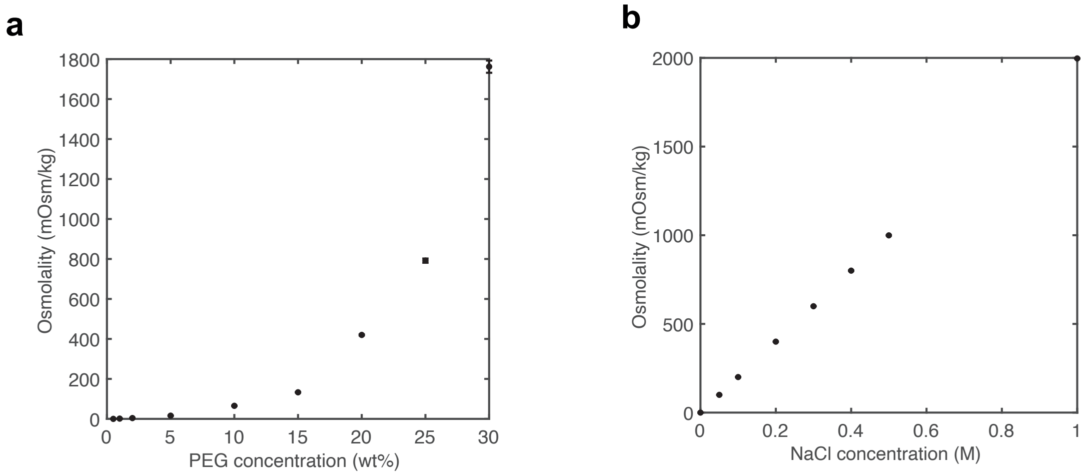

Supplementary Figure 3: Osmolality as a function of NaCl and PEG concentration. Measurements of osmolality for (a) PEG 6000 and (b) salt (NaCl) solution, made with a commercial Fiske Osmometer (Methods), for varying PEG and NaCl concentrations respectively. N=3 for each concentration value. Mean ± SD (error bars are too small to see). Source data are provided as a Source Data file.

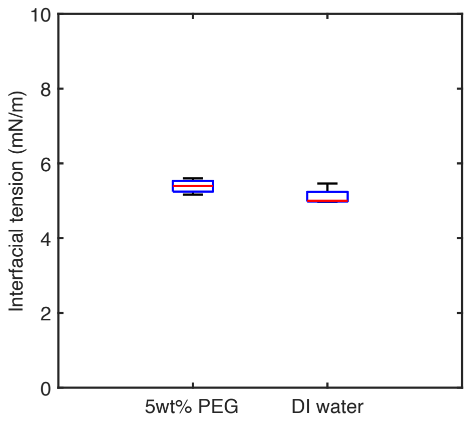

Supplementary Figure 4: Interfacial tension at drop interfaces. Interfacial tension values for HFE7700 with 2% w/w KP600 in DI water (outer droplet interface) and in an aqueous solution of PEG6000 (5% w/w) in DI water (inner droplet interface). N=4 and 5, respectively. Boxplot show Median, 25th and 75th percentiles, whiskers extend to extreme data points. Source data are provided as a Source Data file.

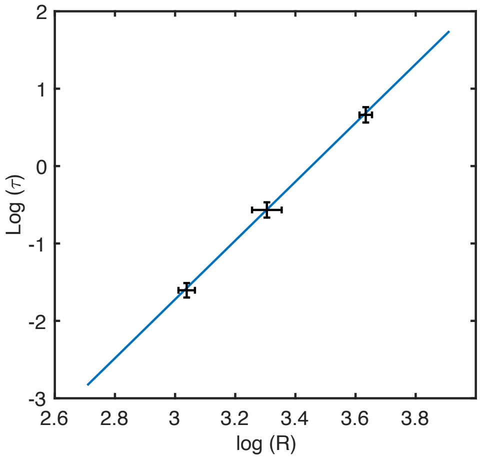

Supplementary Figure 5: Power law dependence of droplet equilibration time with droplet radius. Log-log plot of the characteristic time, τ, as a function of the initial radius of the inner droplet, R, showing that the data follows a power law dependence (linear in log-log scale). Linear fit (blue) was used to obtain the exponent of the power law ($y = a\,x + b$, with a = 3.8 $\pm$ 0.5 and b = -13.1 $\pm$ 2). Mean $\pm$ SD (both in x and y). N = 20, 20, 47 droplets (from left to right). Source data are provided as a Source Data file.

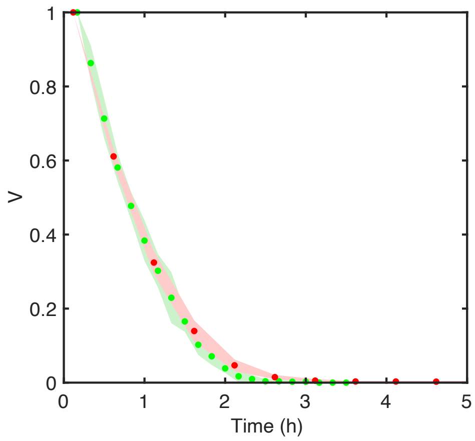

Supplementary Figure 6: FCy5 dye does not significantly affect relaxation kinetics. Temporal evolution of the normalized inner droplet volume V in a 0.4M NaCl solution both in the presence (0.025 mM; green; N=5) and absence (red; N=3) of FCy5 dye in the fluorocarbon oil phase. Initial average droplet radius is 43 µm in both cases. Mean ± SD (shown as error band). Source data are provided as a Source Data file.

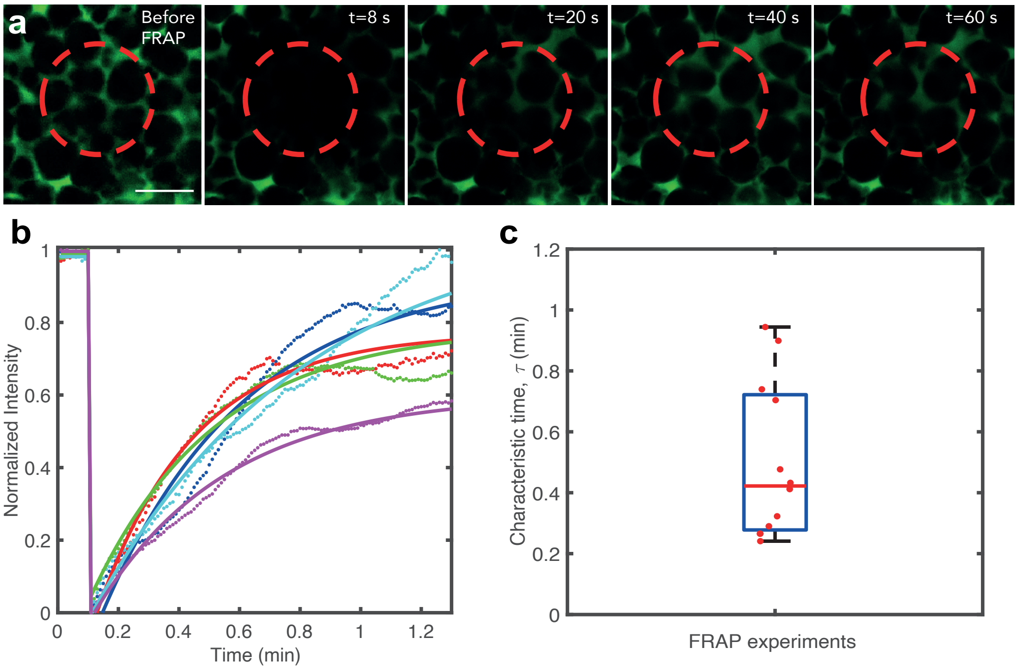

Supplementary Figure 7: Fluorescence recovery after photobleaching (FRAP) of Dextran signal in the interstitial spaces of a developing zebrafish embryo. a, Representative confocal timelapse of an optical section of the zebrafish embryo at sphere stage during and after photobleaching, showing the dynamics of GFP-Dextran (green) in the interstitial fluid between cells. A region of interest (dashed red line) was defined to photobleach the GFP-Dextran and monitor its recovery. Scale bar, 20 μm. **b**, Measured GFP-Dextran intensity profile (dots) as a function of time during FRAP experiment. Distinct colors indicate different experiments. Intensity fluctuations arise from cell rearrangements. Exponential fit (solid lines) were used to obtain the characteristic recovery timescale. **c**, Measured values of the characteristic fluorescence recovery timescale. Each point represents an independent experiment (N=11). Median characteristic time is 0.43 min. Boxplot show Median, 25th and 75th percentiles, whiskers extend to extreme data points. Source data are provided as a Source Data file.
