# Supplementary material for: In situ quantification of osmotic pressure within living embryonic tissues
Source: Nat Commun. 2023 Nov 2;14:7023. doi: 10.1038/s41467-023-42024-9 (PMC10622550; doi:10.1038/s41467-023-42024-9)
Supplement: Supplementary file 3 — Reporting Summary [file 41467_2023_42024_MOESM3_ESM.pdf]

nature portfolio

Corresponding author(s): Otger Campas

Last updated by author(s): Aug 31, 2023

Reporting Summary

Nature Portfolio wishes to improve the reproducibility of the work that we publish. This form provides structure for consistency and transparency in reporting. For further information on Nature Portfolio policies, see our Editorial Policies and the Editorial Policy Checklist.

Statistics

For all statistical analyses, confirm that the following items are present in the figure legend, table legend, main text, or Methods section.

n/a	Confirmed	
☐	☒	The exact sample size (*n*) for each experimental group/condition, given as a discrete number and unit of measurement
☒	☐	A statement on whether measurements were taken from distinct samples or whether the same sample was measured repeatedly
☒	☐	The statistical test(s) used AND whether they are one- or two-sided<br>*Only common tests should be described solely by name; describe more complex techniques in the Methods section.*
☒	☐	A description of all covariates tested
☒	☐	A description of any assumptions or corrections, such as tests of normality and adjustment for multiple comparisons
☐	☒	A full description of the statistical parameters including central tendency (e.g. means) or other basic estimates (e.g. regression coefficient) AND variation (e.g. standard deviation) or associated estimates of uncertainty (e.g. confidence intervals)
☒	☐	For null hypothesis testing, the test statistic (e.g. *F*, *t*, *r*) with confidence intervals, effect sizes, degrees of freedom and *P* value noted<br>*Give P values as exact values whenever suitable.*
☒	☐	For Bayesian analysis, information on the choice of priors and Markov chain Monte Carlo settings
☒	☐	For hierarchical and complex designs, identification of the appropriate level for tests and full reporting of outcomes
☒	☐	Estimates of effect sizes (e.g. Cohen's *d*, Pearson's *r*), indicating how they were calculated

Our web collection on statistics for biologists contains articles on many of the points above.

Software and code

Policy information about availability of computer code

Data collection	Commercial Zeiss Zen software (Zeiss Zen 2012 sp5) was used to perform confocal imaging
Data analysis	Custom software developed in Matlab (Mathwork , version 2021b) was used to analyze droplet sizes. It can be found on GitHub at: https://github.com/campaslab/Osmotic-Pressure-droplet-volume-reading. Matlab (Mathwork , version 2021b) and ImageJ (v1.53i) were also used to analyze the data

For manuscripts utilizing custom algorithms or software that are central to the research but not yet described in published literature, software must be made available to editors and reviewers. We strongly encourage code deposition in a community repository (e.g. GitHub). See the Nature Portfolio guidelines for submitting code & software for further information.

Data

Policy information about availability of data

All manuscripts must include a data availability statement. This statement should provide the following information, where applicable:

- Accession codes, unique identifiers, or web links for publicly available datasets
- A description of any restrictions on data availability
- For clinical datasets or third party data, please ensure that the statement adheres to our policy

All data supprting our findings are provided with this manuscript as source data files

Research involving human participants, their data, or biological material

Policy information about studies with human participants or human data. See also policy information about sex, gender (identity/presentation), and sexual orientation and race, ethnicity and racism.

Reporting on sex and gender	N/A
Reporting on race, ethnicity, or other socially relevant groupings	N/A
Population characteristics	N/A
Recruitment	N/A
Ethics oversight	N/A

Note that full information on the approval of the study protocol must also be provided in the manuscript.

Field-specific reporting

Please select the one below that is the best fit for your research. If you are not sure, read the appropriate sections before making your selection.

☒ Life sciences ☐ Behavioural & social sciences ☐ Ecological, evolutionary & environmental sciences

For a reference copy of the document with all sections, see nature.com/documents/nr-reporting-summary-flat.pdf

Life sciences study design

All studies must disclose on these points even when the disclosure is negative.

Sample size	In experiments involving zebrafish embryos, no statistical methods were used to pre-determine sample sizes but our sample sizes are similar to those reported in previous publications (Refs. 9, 32, 65)
Data exclusions	No data was excluded
Replication	We have indicated in the manuscript when (and how many times) specific experiments were independently replicated
Randomization	No randomization of the data was used because if not relevant to this study
Blinding	Analysis was done by automated software which was blind to data collection. Team members doing the analysis were different from those who collected the data and were blinded to group allocation

Reporting for specific materials, systems and methods

We require information from authors about some types of materials, experimental systems and methods used in many studies. Here, indicate whether each material, system or method listed is relevant to your study. If you are not sure if a list item applies to your research, read the appropriate section before selecting a response.

Materials & experimental systems

n/a	Involved in the study	
☒	☐	Antibodies
☒	☐	Eukaryotic cell lines
☒	☐	Palaeontology and archaeology
☐	☒	Animals and other organisms
☒	☐	Clinical data
☒	☐	Dual use research of concern
☒	☐	Plants

Methods

n/a	Involved in the study	
☒	☐	ChIP-seq
☒	☐	Flow cytometry
☒	☐	MRI-based neuroimaging

Animals and other research organisms

Policy information about studies involving animals; ARRIVE guidelines recommended for reporting animal research, and Sex and Gender in Research

Laboratory animals	Zebrafish (Danio rerio) embryos were used in this study. Only embryos up to 5 hours post fertilization were used
Wild animals	None
Reporting on sex	Sex experiments were not necessary, as zebrafish embryos at the studies stage have not yet undergone sex determination
Field-collected samples	None
Ethics oversight	Experiments were performed following all ethical regulations and according to protocols approved by the Institutional Animal Care and Use Committee (IACUC) at the University of California, Santa Barbara

Note that full information on the approval of the study protocol must also be provided in the manuscript.
